# The Roles of Glutamate Receptors and Their Antagonists in Status Epilepticus, Refractory Status Epilepticus, and Super-Refractory Status Epilepticus

**DOI:** 10.3390/biomedicines11030686

**Published:** 2023-02-23

**Authors:** Tzu-Hsin Huang, Ming-Chi Lai, Yu-Shiue Chen, Chin-Wei Huang

**Affiliations:** 1 Department of Neurology, National Cheng Kung University Hospital, College of Medicine, National Cheng Kung University, Tainan 70142, Taiwan; 2 Zhengxin Neurology & Rehabilitation Center, Tainan 70459, Taiwan; 3 Department of Pediatrics, Chi-Mei Medical Center, Tainan 71004, Taiwan

**Keywords:** status epilepticus, NMDA, AMPA, ketamine, perampanel

## Abstract

Status epilepticus (SE) is a neurological emergency with a high mortality rate. When compared to chronic epilepsy, it is distinguished by the durability of seizures and frequent resistance to benzodiazepine (BZD). The Receptor Trafficking Hypothesis, which suggests that the downregulation of γ-Aminobutyric acid type A (GABAA) receptors, and upregulation of N-methyl-D-aspartate (NMDA) and α-amino-3-hydroxy-5-methyl-4-isoxazolepropionic acid (AMPA) receptors play major roles in the establishment of SE is the most widely accepted hypothesis underlying BZD resistance. NMDA and AMPA are ionotropic glutamate receptor families that have important excitatory roles in the central nervous system (CNS). They are both essential in maintaining the normal function of the brain and are involved in a variety of neuropsychiatric diseases, including epilepsy. Based on animal and human studies, antagonists of NMDA and AMPA receptors have a significant impact in ending SE; albeit most of them are not yet approved to be in clinically therapeutic guidelines, due to their psychomimetic adverse effects. Although there is still a dearth of randomized, prospective research, NMDA antagonists such as ketamine, magnesium sulfate, and the AMPA antagonist, perampanel, are regarded to be reasonable optional adjuvant therapies in controlling SE, refractory SE (RSE) or super-refractory SE (SRSE), though there are still a lack of randomized, prospective studies. This review seeks to summarize and update knowledge on the SE development hypothesis, as well as clinical trials using NMDA and AMPA antagonists in animal and human studies of SE investigations.

## 1. Introduction

Status epilepticus (SE) is a potentially fatal neurological condition. SE, in contrast to simple seizure disorders, has various mechanisms for sustaining seizures, often resulting in irreversible neuronal damage. Early clinical data imply that the longer the seizures last, the more resistant they become to treatment [1].

Seizures become unresponsive to routinely used antiepileptic drugs (AEDs) and benzodiazepines, as they progress toward unmanageable conditions, a condition known as refractory status epilepticus (RSE) [2]. RSE escalates over time to a more hazardous condition known as super-refractory status epilepticus (SRSE), in which even constantly injected anesthetic agents fail to cease the electrical discharge, and neuronal death is inevitable [3].

In the central nervous system (CNS), glutamate serves as the main neurotransmitter in excitatory function. N-methyl-D-aspartate (NMDA) receptors and α-amino-3-hydroxy-5-methyl-4-isoxazolepropionic acid (AMPA) receptors are two groups of the ionotropic glutamate receptors (iGluRs) family [4], which are extensively distributed in the CNS and are necessary in maintaining normal brain function and are thought to be involved in many neuropsychiatric diseases, including cerebral ischemia, traumatic brain injury, Alzheimer’s disease, Huntington’s disease, Parkinson’s disease, epilepsy, neuropathic pain, depression, autism, and schizophrenia [5].

The underlying mechanism of SE has long been studied [6]. Currently, the downregulation of GABAA receptors and upregulation of NMDA and AMPA receptors via transmission and membrane distribution are key to the process of SE maintenance and progression, which is called the Receptor Trafficking Hypothesis [7,8]. Based on this hypothesis, NMDA, AMPA receptor antagonists and modulators have been evaluated in vitro and in vivo for their neuroprotective and anticonvulsant effects, which are significant in various animal SE models [9,10,11]. While in human studies, many of these antagonists, such as phencyclidine (PCP) and MK-801, demonstrated profound psychotomimetic effects due to the widespread distribution of these receptors and their roles in maintaining normal brain function, this led to their failure to be approved as clinically therapeutic regimens [5].

Overall, the literature review aims to summarize prior understanding of NMDA and AMPA receptors, as well as their associations with SE, RSE, and SRSE. Furthermore, all therapeutic trials involving AMPA and NMDA receptor antagonists associated with SE will be included.

## 2. Materials and Methods

Articles from PubMed, MEDLINE, Cochrane Library, clinicaltrials.gov, and reference lists of pertinent papers were scoured. These articles were published in English before December 2022. Original articles, case reports, clinical trials, reviews, meta-analyses, and systematic reviews are all examples of articles. We rejected letters because there was an insufficient amount of information. The main search terms were “NMDA,” “AMPA,” “status epilepticus,” “refractory status epilepticus,” and “super-refractory status epilepticus.” We omitted phrases such as “encephalitis” and “autoimmune” to focus on SE in adults.

**Figure 1:**
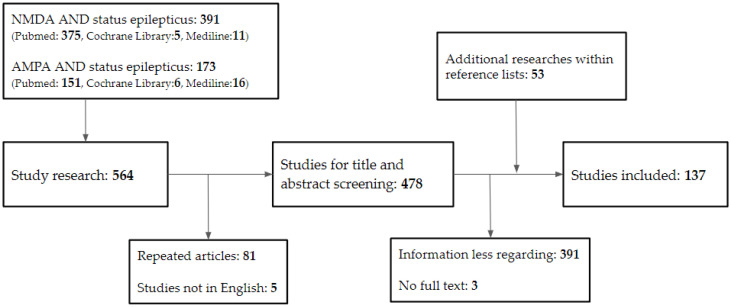
Database search flowchart. NMDA, N-methyl-D-aspartate; AMPA, α-amino-3-hydroxy-5-methyl-4-isoxazolepropionic acid.

Following the removal of duplicates from the search results, the titles and abstracts of the remaining studies were reviewed for potential eligibility. Following that, full texts of the publications recommended for inclusion were screened, and their references were checked for any further research that was required. Articles that did not discuss the relationship between NMDA, AMPA, and SE were excluded. The review contained 137 papers in total (Figure 1).

## 3. Fundamental Roles of NMDA and AMPA Receptors

Glutamate and gamma aminobutyric acid (GABA) in the CNS serve as the main neurotransmitters in excitatory and inhibitory function, respectively. Glutamatergic neurons compose nearly 70–80% of neurons in the cerebral cortex, with GABAergic interneurons composing the remainder [12]. Glutamate receptors can be split into two groups: ionotropic glutamate receptors (iGluRs) that serve as ligand-gated ion channels and metabotropic glutamate receptors (mGluRs) as the members of the G protein-coupled receptor superfamily [13]. Moreover, iGluRs can be split into three main receptor families designated after their prototypic agonists: NMDA, α-amino-3-hydroxy-5-methyl-4-isoxazolepropionic acid (AMPA), and kainate (KA).

Previously, glutamate receptor agonists such as domoate were known to induce seizures both in animals [14] and humans [15], indicating the importance of glutamate receptors in the field of epilepsy. Furthermore, NMDA and AMPA have been shown to cause seizures in rodent models [16,17], and antagonists against their receptors exhibit anticonvulsant actions. According to the findings, glutamate receptors, particularly ionotropic glutamate receptors, may play a substantial role in seizure genesis. Following clinical trials on NMDA antagonists, AMPA antagonists have also demonstrated their antiseizure effects.

### 3.1. NMDA Receptors

As a member of the ionotropic glutamate receptor family, the NMDA receptor has been widely studied [18], and it has been discovered to be involved in maintaining brain function, neurodevelopment [19], learning, and memory formation [20,21]. NMDA receptors are distinguished from other iGluRs by their high Ca^2+^ ion permeability and voltage-dependent channel blockade [5]. They are primarily found in the CNS in the postsynaptic membrane where they interact with AMPA receptors but are also expressed in presynaptic and extrasynaptic membranes with lower densities [22]. When compared to AMPA receptors, these receptors activate and decay far more slowly [23].

The NMDA receptor is an ion channel permeable for calcium, composed of four subunits derived from three gene families: GluN1, GluN2A-D, and GluN3A-B. Each receptor is a tetramer with either two GluN1 plus two GluN2 subunits, or two GluN1 plus two GluN3 subunits [24,25]. The activation of NMDA receptors requires the presence of the agonist glutamate, along with either glycine or D-serine as a co-agonist [26]. In contrast to AMPA receptors, the NMDA ion channel has a voltage-dependent blocking by Mg^2+^ at the resting membrane potential level [27]. This ion channel requires membrane depolarization to remove the magnesium block for Ca^2+^, Na^+^, and K^+^ ions to pass through [24]. Under diverse situations, the intracellular Ca^2+^ influx initiates several downstream signal processes in the postsynaptic neurons, which serve physiological or pathophysiological purposes [28]. Over-activation of NMDA receptors has been proven to generate excessive Ca^2+^ influx into neurons, resulting in cellular damage and death [29].

While excitatory amino acid transporters keep glutamate concentrations low, it has been discovered that higher glutamate emerges in the epileptic area relative to control regions in patients with epilepsy [30] and animal models of epilepsy [31]. These circumstances are perfect for prolonging the prolonged activation of NMDA receptors in SE. The activation of NMDA receptors has two implications [6]. Firstly, it would cause prolonged depolarization, allowing more NMDA channels to open. Secondly, it causes intracellular calcium buildup, which may mediate apoptotic programmed cell death via osmotic stress and other mechanisms [32].

### 3.2. AMPA Receptors

The AMPA receptors are located largely postsynaptic, directly activated by the binding of glutamate, and they mediate most of the fast excitatory synaptic transmission in the mammalian CNS. AMPA receptors are heterotetramers composed of four kinds of subunits: GluA1, GluA2, GluA3, and GluA4, which work together to form an ion channel. GluA2 is assumed to be critical for controlling channel rectification and ion permeability [33]; hence, many researchers have focused on GluA2 subunit expression levels. AMPA receptor Ca^2+^ conductance varies depending on the presence of the GluA2 subunit in the tetramer complex [34]. GluA2-lacking AMPA receptors are typically Ca^2+^-permeable (CP). These CP-AMPA receptors are primarily unusual, whereas mature brains normally contain GluA2-containing AMPA receptors that are Ca^2+^-impermeable (CI) [35]. The presence of the GluA2 subunit in AMPA receptors limits the influx of Ca^2+^ and Zn^2+^.

Seizures, according to animal research, can produce rapid dynamic alterations in the AMPA receptor subunit composition and function [33]. These studies found that AMPA receptor transmission during SE is related to increased expression of GluA1 and decreased expression of the GluA2 subunit in the surface membrane [36,37]. Ca^2+^ influx-triggered excitotoxicity via Ca^2+^-permeable AMPA receptors has also been shown in several illness models [34].

To summarize, the NMDA and AMPA receptors play an important role in the maintenance and development of normal neural networks in the CNS by controlling the permeability of ions, primarily Ca^2+^, and may be capable of initiating seizures and maintaining seizures in SE, which leads to further neuronal damage or death.

## 4. SE

SE is a neurological emergency with a significant mortality and morbidity rate, depending on the patients’ ages, underlying diseases, and etiologies [38]. SE is defined as “...a condition caused by the failure of mechanisms for seizure termination or the initiation of mechanisms that result in excessively extended seizures (after time point t1). It is a disorder that can have long-term effects (after time point t2), such as neuronal death, neuronal injury, and alteration of neuronal networks…”, stated the International League Against Epilepsy (ILAE) in 2015 [2]. The time points t1 and t2 in the case of convulsive SE are assumed to be 5 min and 30 min, respectively, based on animal and clinical investigations [2].

Various routes lead to seizure [39], with the imbalance between neuron excitation and inhibition being commonly regarded to be the most important. However, because the underlying mechanism of SE differs from that of a simple seizure, the potential therapy and results are not the same.

The most distinguishing feature of SE is its self-sufficiency and increased resistance to benzodiazepines over time. Animal and clinical research in 1997 and 1998 revealed that benzodiazepines are efficacious early in the course of SE, but lose 20-fold potency by 30 min, and fail to halt SE by 45 min [40,41,42]. Retrospective research on the duration of convulsive SE found that first-line therapy was beneficial in 80% of patients when given within 30 min of seizure onset, and then it dropped to no more than 40% beyond 2 h [43].

### 4.1. Receptor Trafficking Hypothesis

The Receptor Trafficking Hypothesis, a generally accepted theory of self-sustaining seizures in SE, was developed. This hypothesis aims to explain the gradual resistance to GABAA receptor modulators and progressive sensitivity to NMDA antagonists by claiming that decreased expression of GABAA receptors and increased expression of NMDA receptors in the postsynaptic membrane cause the progression [44].

Several studies published in the 1990s suggested the reduced effects of GABA-mediated inhibition during SE due to fast GABARs alterations [45,46]. The phenomenon of GABAA receptor internalization and decreasing density in the postsynaptic membrane was then reported for the first time in 2005 [47,48]. One study used cultures of hippocampal pyramidal neurons undergoing recurrent bursting and found that increased neuronal activity accelerated GABAA receptor internalization, whereas blocking neuronal activity reduced it [47]. Another study utilizing dentate hippocampal cells found that after 1 h of in vivo SE, the number of functional GABAA receptors in the synapse decreased by 50% [48].

**Figure 2:**
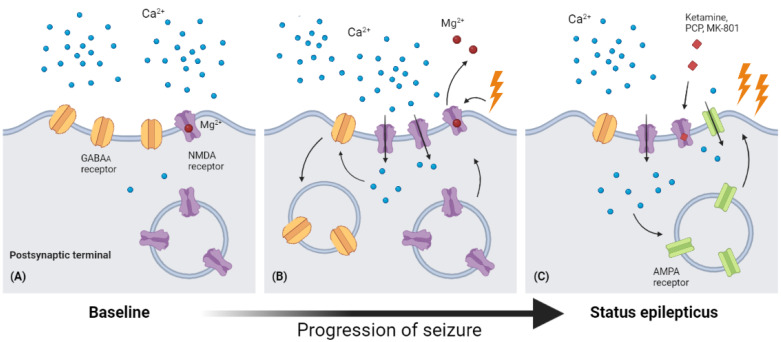
Receptor traffic and modulation during the process of SE. (**A**) In baseline conditions, Mg^2+^ blocks the ion channel of the NMDA receptor, leading to low permeability of Ca^2+^. (**B**) As seizure occurs, the depolarization of the postsynaptic membrane would cause Mg^2+^ to leave the binding site in the ion channel, leading to a significantly increased permeability of Ca^2+^. The surging intracellular Ca^2+^ then causes endocytosis of GABAA receptors and up expression of NMDA receptors. (**C**) The increasing intracellular Ca^2+^ level also enhances AMPA expression in the postsynaptic membrane, and this increased expression of NMDA and AMPA receptors may explain the increasing anticonvulsant effects of NMDA receptor antagonists (ketamine, PCP, MK-801) (Figure created with BioRender.com).

NMDA antagonists, on the other hand, keep their impact until the late stages of SE in animal and pediatric investigations, whereas most GABAergic-boosting drugs soon lose their potency during SE [49,50,51]. It is well-known that NMDA receptor trafficking is important in many aspects of neurology, including development [52], degenerative diseases [53,54], and excitotoxicity/cell death [55], but until 2013, a study of dentate gyrus granule and CA3 pyramidal cells demonstrated the relocation of NMDA receptors from the intracellular area to the synaptic surface [56]. Meanwhile, it is predicted that after 1 h of SE induced by lithium–pilocarpine, the number of functional postsynaptic NMDA receptors increases by 38%, and this action also contributes to the augmentation of phasic and tonic excitatory currents during SE (Figure 2).

The activation of NMDA receptors also seems to improve AMPA-mediated transmission during SE and prolonged seizures [36]. There are observations that GluA1-containing AMPA receptors are rapidly inserted in the surface membrane after NMDA receptor activation and Ca^2+^ influx, which induces the phosphorylation of serine 831 and serine 841 in the GluA1 subunit [57,58]. 

### 4.2. Other Factors Related to or Contributing to SE

In addition to GABAA and NMDA receptors, the inactivation of K(+)/Cl(−) cotransporter (KCC2) may contribute to enhanced resistance to benzodiazepines throughout the process of SE. The function of KCC2 is to maintain intracellular Cl- at a low level compared with extracellular concentrations, which is required for effective synaptic regulation of GABAA receptors. In a 2015 animal study [59], phosphorylation of residue S940, which is rapidly dephosphorylated during SE and leads to higher intracellular Cl-, increased the modulation of membrane trafficking and transport activity of KCC2, which decreases the neuronal inhibition function carried by GABAA receptors. According to another model [60], the compromising activities of KCC2 will result in longer and more severe seizure episodes.

Several antagonists for NMDA receptors and AMPA receptors were investigated in clinical research, according to the findings above. However, the majority of NMDA receptor antagonists have not been shown to be efficacious or safe for clinically therapeutic use, and only one AMPA receptor antagonist, perampanel, has been shown to treat multiple kinds of epilepsy. The following sections will go into greater detail about these investigations.

### 4.3. RSE and SRSE

If SE is not treated or delayed beyond five minutes, it may proceed to RSE or SRSE. RSE is defined as SE that persists despite appropriate doses of an initial benzodiazepine followed by a second AED. It is time to consider initializing continuous anesthetic medications, such as midazolam, propofol, pentobarbital, and ketamine [61]. Furthermore, SRSE is a severe form of RSE that was first introduced at the Third London–Innsbruck Colloquium on SE held in Oxford on 7–9 April 2011 [62], with the definition of persisting for 24 h or more despite treatment with continuous third-line anesthetic agents, including cases that recur after reducing or withdrawing the anesthesia.

In a 2017 Germany health insurance database analysis, SRSE accounted for approximately 13% of all SE patients, with a substantially higher mortality rate (nearly 40%) when compared to 15% of RSE and 9.6% of SE [63]. In this database, the incidence rate of SRSE is 3.0 per 100,000 per year, compared to 15.0 in 100,000 for SE. Overall, RSE accounts for 12–48% of SE cases, while SRSE accounts for 3–10% of SE patients [64,65]. In published investigations, RSE mortality varies from 10% to 40% and SRSE mortality ranges from 35% to 65% [63,66].

RSE and SRSE appear to be more SE, but their epidemiologies differ from those of non-refractory SE; they are more likely to suffer significant brain insults such as trauma, infection, or stroke than as a result of chronic epilepsy [64,67]. It is not rare for RSE to emerge de novo in a previously completely healthy person without an obvious precipitant, a condition known as new-onset RSE, or NORSE [68], which was first used in 2005. The treatment strategy for NORSE is the same in the first 2–3 days after the seizure onset to manage the SE, but a rapid diagnostic workup is required to rule out any probable infection, and to initiate immune therapies [69]. Although the terms RSE and SRSE were used to characterize a condition with a homogeneous group of people, it is more likely that the underlying causes are quite heterogeneous as medical knowledge progresses. Furthermore, there is probably a shared pathway leading to a single mechanism sustaining seizures in SE patients, which could serve as a future treatment target.

#### Sudden Unexpected Death in Epilepsy (SUDEP)

People with epilepsy face a higher mortality rate than the general population [70], and may die from several causes including aspiration of stomach contents, suffocation, falls, motor vehicle accidents, suicide, and SUDEP. Among these causes, SUDEP is the leading cause of death in patients with epilepsy, especially in patients with refractory epilepsy [71]. The most widely used definition of SUDEP currently is “the sudden, unexpected, witnessed or unwitnessed, nontraumatic, and nondrowning death in patients with epilepsy, with or without evidence for a seizure, and excluding documented SE, in which postmortem examination does not reveal a toxicological or anatomical cause for death” [72].

SUDEP is often found to occur at night during sleep with a prone position, and is thought to be related to an impairment in controlling respiratory, cardiac, and arousal function in the brainstem [73,74]. Apnea and bradycardia progressing to asystole and death in the ictal or postictal period are considered as contributing to SUDEP [73]. 

Though SE has been excluded in the definition of SUDEP, their relationships with cardiorespiratory dysfunction might share similar mechanisms of triggering asystole and apnea. In the possible underlying mechanism of SUDEP, medullary spreading depolarization (SD) is related to the brainstem dysfunction in the mice SUDEP model [75]. Interestingly, mice pretreated with NMDA receptor antagonists (MK-801 or memantine hydrochloride) showed profound prevention of mortality and brainstem SD [75]. In another rat seizure model using intrahippocampal 4-aminopyridine (4-AP), SE with intermittent brainstem seizure events could cause cardiorespiratory depression that leads to death, as compared to SE without brainstem seizure causing increased respiratory and heart rates [76]. 

Future studies may need to investigate the effects of NMDA antagonists in reducing the occurrence of SUDEP or SE related cardiorespiratory events, and help to develop preventive strategies. 

### 4.4. NMDA and Its Antagonists in SE, RSE, SRSE

The first NMDA antagonists were broad-spectrum ion channel blockers that had a considerable psychotomimetic impact due to the widespread unselective blockade of NMDA receptors across the CNS [27]. For their authorizations, many of them were halted or withdrawn. Some non-competitive ion channel blockers with lower affinity are now Food and Drug Administration (FDA)-approved for human usage, including dextromethorphan, ketamine, esketamine, memantine, and amantadine [5]. So far, research has focused on a wide range of neuropsychiatric diseases, including cerebral ischemia, traumatic brain injury, Alzheimer’s disease, Huntington’s disease, Parkinson’s disease, epilepsy, neuropathic pain, depression, autism, and schizophrenia [5].

In contrast to regulating long-term epilepsy, antiepileptic medicines used for SE have rising or diminishing efficacy as the process of SE progresses [41,50]. In SE animal models and subsequent clinical trials, it is known that NMDA receptor antagonists have increased efficacy in stopping seizures. Even though several NMDA receptors have been explored, few of them have acceptable side effects to be approved as therapeutic agents [5].

NMDA receptor channel blockers and modulators; glutamate and glycine site antagonists; positive and negative allosteric modulators; and compounds acting downstream of NMDA receptors are currently available [77]. However, the classification is constantly modified by new evidence from clinical trials or newly discovered molecules. The following sections mainly describe some antagonists linked with SE (Table 1).

#### 4.4.1. Ketamine

Ketamine is a non-competitive NMDA antagonist with low affinity at the phencyclidine site within the NMDA receptor ion channel, which helps to shorten channel opening time [78]. It is a derivative of phencyclidine (PCP), which is a safe anesthetic but frequently causes prolonged delirium and sensory deprivation during the postoperative recovery period. Ketamine was initially synthesized in 1962, named CI-581 at that time, and was widely utilized for surgical anesthesia in the Vietnam War due to its lower potency, rapid onset, and short half-life, making it a much more appealing option to dizocilpine (MK-801) [79] and PCP. However, a distinct effect known as “dissociative anesthesia” was discovered [80], in which people appear awake, retain the laryngeal reflex, and are capable of protecting their airways but are unable to respond to sensory stimuli, raising concerns about the medication and leading to a decline in its use as a human anesthetic. Between the 1970s and 1990s, ketamine fell out of the medical community and was widely used as a recreational drug due to its “psychedelic effect” in subanesthetic doses [81], becoming a schedule III drug.

Ketamine regained its attention in medical use around the 1990s as more data concerning its mechanism of action and efficacy became available [82]. Ketamine has been established in anesthetic studies to be a safe and effective option for treating postoperative pain and neuropathic pain. Additionally, its neuromodulation effect makes it a candidate for treating multiple neurological diseases, such as depression [83], post-traumatic stress disorder [84], subarachnoid hemorrhage (SAH) [85], traumatic brain injury (TBI) [86], migraine [87], and refractory SE.

Ketamine has long been shown to have anticonvulsant activities in animal studies [21]. In the following clinical studies, ketamine was examined in RSE and SRSE patients, due to its higher efficacy in the late phase of SE in animal models. In a 2020 single-center retrospective study [88], 68 SRSE patients were treated with ketamine plus midazolam infusion, which resulted in at least 50% seizure burden in 81% of patients, and complete cessation in 63%. Its results suggest that ketamine did not affect ICP even at prolonged anesthetic dosages, as do the results of a recent comprehensive analysis on non-traumatic neurological illness [89], and decreased vasopressor requirement. A 2022 prospective study with 11 patients with SRSE found lesser permanent control of SE (27%) compared to earlier retrospective studies (28–96%) [90]. Due to patient selection bias, this study may suggest that the efficacy of ketamine is not as good as other studies have demonstrated.

Ketamine, on the other hand, is considered an optional therapy due to its favorable safety profile [91,92]. Some advantages of ketamine over conventional anesthetic agents include less cardiovascular suppression effect and prevention of endotracheal intubation [93]. These properties make them promising options for managing SE or nonconvulsive SE in critically ill patients [94].

Overall, future multicenter randomized controlled trials must define the appropriate timing and dosage of ketamine utilized in SE or further refractory SE. KETASER01, a multicenter, randomized, controlled, open-label clinical trial was discontinued in 2020, owing to its low patient eligibility and unsuccessful recruitment [95]. It attempted to evaluate the efficacy of ketamine to that of conventional anesthetics in children with refractory convulsive status epilepticus. Future clinical research on similar topics may include more centers, with funding to participants, and more feasible protocols to avert possible failure. 

#### 4.4.2. Magnesium Sulfate

Magnesium sulfate has been used to treat eclamptic seizures since first reported in 1916 [96], and it is still the most commonly used medication in obstetric practice today [97]. Intravenous doses achieve therapeutic levels almost immediately, and its only FDA-approved indication in obstetrics is for the prevention or treatment of eclampsia [97].

Mg^2+^ has been proven to have an antiepileptic impact in animal research, and this has been known for decades [98]. Its effects are validated mostly by blocking the NMDA receptor’s ion channel to prevent the Ca^2+^ from passing through [99]. Early studies identified the potential for convulsion in the Mg^2+^ depletion state [100]. 

In a major randomized controlled trial, magnesium sulfate was proven effective in controlling SE due to eclampsia [101] and became the first-line therapy in this condition. However, the evidence level of intravenous magnesium sulfate administration for non-eclamptic SE/RSE is Oxford level 4 Grade D, and routine use of MgSO4 in these patients is not suggested until additional prospective studies establish its efficacy [102].

#### 4.4.3. MK-801 (Dizocilpine)

MK-801 was discovered in 1982 as a selective, non-competitive antagonist of NMDA receptors. Its binding site is in the ion channel of NMDA receptors, inhibiting the flow of ions, primarily Ca^2+^, through the channel. MK-801 efficiently stopped SE and reduced the death rate when paired with diazepam in both lithium–pilocarpine and soman-induced SE animal studies [103,104].

The development of MK-801 as a therapeutic agent was halted, mainly due to its side effect of the strict ON/OFF mechanism of NMDA receptor blockade in preclinical trials [105,106]. Nevertheless, it is still commonly used in animal SE models as a comparative medication or research tool.

#### 4.4.4. Amantadine

Amantadine is a low-affinity non-competitive NMDA receptor antagonist. It primarily increases dopamine release while decreasing dopamine reuptake in the CNS. Amantadine was shown to have an NMDA receptor blockade effect by increasing the rate of channel closure in 1991 [107].

There have been few studies on the efficacy of amantadine in SE. A retrospective investigation on electrical status epilepticus in sleep (ESES) discovered that amantadine may influence ESES-associated syndrome, and may have benefits on cognitive function and behavior [108].

#### 4.4.5. Memantine

Memantine, an amantadine derivative, is a non-competitive NMDA receptor antagonist that binds to or near Mg^2+^ binding sites [109]. Memantine has been demonstrated to preferentially operate on the extrasynaptic NMDA receptors [110], where over-activation has been associated with neurodegeneration in AD. As a result, memantine has been licensed for use as a therapeutic drug in AD [111]. 

Memantine has demonstrated the potential to prevent cognitive deficits in numerous animal seizure models and appears to have a considerable neuroprotective effect against glutamate and NMDA neurotoxicity [112,113]. Memantine appears to diminish neurotoxicity in pilocarpine-induced SE and convulsion duration in pentylenetetrazole (PTZ) models [113,114]. There are no current studies on seizures or SE with memantine.

#### 4.4.6. Dextromethorphan

Dextromethorphan is among the most commonly used cough suppressants and FDA-approved NMDA receptor channel blockers for more than 50 years [115]. Dextromethorphan has displayed some efficacy against seizures in multiple animal models [116]. A few human investigation cases and case reports have been noted, with possible effects in managing refractory epilepsy due to brain damage or partial epilepsy [117,118].

Although there are numerous NMDA receptor modulators not only on the NMDA receptor itself but also on the downstream pathway, only a handful have the potential to be beneficial in alleviating seizures. NMDA receptor antagonists, such as ifenprodil, felbamate, remacemide, and riluzole have been proven to have some effect in animal seizure models but have not been conducted in SE models.

### 4.5. AMPA and Its Antagonists in SE, RSE, SRSE

Preclinical experiments for the antiseizure effects of competitive and non-competitive AMPA receptor antagonists have been conducted [126]. At this time, the FDA has approved one of the AMPA receptor antagonists, perampanel, and several candidates called GYKI52466 and NS1209 have limited therapeutic utility due to the short duration of action and magnitude of adverse effects seen at effective doses [127,128]; so, further studies have been halted (Table 1).

#### Perampanel

Perampanel is a selective, non-competitive AMPA receptor antagonist that has been licensed by the United States FDA for the treatment of partial-onset seizures with or without secondary generalization as monotherapy or adjunctive therapy. Perampanel and GYKI52466 were able to stop pilocarpine-induced severe SE in rats that had resistance to diazepam [129]. In the view of SE in human studies, some evidence came from case reports, case series, and retrospective investigations, and some fresh data are emerging, demonstrating that perampanel is effective and has a satisfactory safety profile in the treatment of RSE and SRSE [130,131].

In 2022, a systematic review gathered twenty-one studies with a total of 369 cases of SE, including 220 cases of RSE, and 70 cases of SRSE. Perampanel was employed in 324 cases with initiation time ranging from 30 min and 59 days after SE onset. A total of 119 cases (36.6%) were considered responders. It highlighted that real-world evidence of perampanel as a potential therapeutic option in SE of any type is rising, but more clinical research is needed to establish the appropriate timing, dosage, and titration that are efficacious and safe for SE cessation [132]. Another retrospective investigation found similar outcomes with the use of perampanel in refractory cases with a response rate of 36.5% [133]. Perampanel is not yet routinely used in the guidelines for treating SE or RSE in America [134] and Europe based on existing evidence from retrospective data [135]. Further randomized control prospective trials are required to achieve the proper dose, timing, and duration of perampanel use in SE patients.

### 4.6. Combined Polytherapy with NMDA and AMPA Antagonists

Several studies have focused on the early use of polytherapy with antiepileptic drugs in different mechanisms to achieve a synergic effect, particularly GABA agonists with NMDA and AMPA antagonists, with the conception of gradual loss of receptor response to benzodiazepines and enhanced sensitivity to glutamate [8].

Studies have displayed some remarkable outcomes in animal SE models. Therapy with phenobarbital, midazolam, and ketamine has been demonstrated to be superior to monotherapy with phenobarbital or midazolam in cholinergic-induced SE [136]. Another study discovered similar results in the lithium–pilocarpine SE model that the midazolam–ketamine–valproate combination therapy based on the receptor trafficking theory was considerably more effective than the midazolam–fosphenytoin–valproate combination utilized in clinical guidelines [137].

In human studies, according to clinicaltrials.gov, there are no active or published trials in SE regarding NMDA and AMPA combination therapy. Future clinical trials may need to incorporate an early polytherapy arm to administer treatment as soon as the patient is recognized to be RSE.

### 4.7. Limitation 

This literature review focused on the NMDA and AMPA antagonists in the treatment of SE, RSE, SRSE, in which NMDA and AMPA antagonists may demonstrate good efficacy [50]. Despite their promising pharmacologic characteristics, their effects on behavior and cognitive function still need special attention due to their wide distribution, and the crucial roles of NMDA and AMPA receptors in the CNS [12,19]. Moreover, the interaction of these antagonists with other standard AEDs in polytherapy, when treating SE, requires further investigations in the future. 

## 5. Conclusions

The complete control of SE or RSE is challenging; although, newer generations of antiepileptic drugs/antiseizure medications have been developed. Because of their unique function in the development of SE, NMDA receptor antagonists such as ketamine and magnesium sulfate, as well as AMPA receptor antagonists such as perampanel, currently present potential synergistic effects with benzodiazepine and conventional AEDs. Further prospective, randomized studies about the appropriate timing, dosage, and duration of these antagonists are needed to reduce the incidence of SRSE and to achieve early seizure control to prevent long-term neurotoxicity.

## Figures and Tables

**Table 1:** The various compounds (NMDA and AMPA antagonists), their trials on SE, and characteristics.

Receptor	Name	Mechanism	Binding Site	Clinical Trial in SE	Additional Characteristics
NMDA	Ketamine	non-competitive antagonist	PCP site	Retrospective studies have shown their potential effects as adjuvant drugs. Randomized and prospective studies are needed	- Rapid onset, short half-life, and fewer hypotension events in critical illness patients - Most side effects of ketamine are dose-dependent, transient, and self-resolving. Deaths caused by ketamine overdose, in the absence of multidrug intoxication, are very rare [119]
	Magnesium Sulfate	non-competitive antagonist	Mg^2+^ site	Significantly effective for eclamptic SE. No solid evidence was found in non-eclamptic SE	- Be aware of areflexia of the patellar deep tendon reflex, followed by respiratory paralysis or cardiac arrest as the plasma level of Mg^2+^ increases [120] - Magnesium-induced vasodilation is suspected due to its action on most types of calcium channels in vascular smooth muscle, leading to decreased intracellular calcium and smooth muscle relaxation [120]
	phencyclidine(PCP)	non-competitive antagonist	PCP site	No clinical studies due to its severe emergence phenomena, which is characterized by euphoria, vivid dreams, illusions, and hallucinations [121]	- PCP was widely abused since 1965 with schizophrenia-like symptoms which may persist for weeks after its last use, and some abusers progressed to schizophrenia later - Behavioral changes documented in animal studies, and presumably in humans, are indicated to occur through PCP binding site antagonism
	MK-801 (Dizocilpine)	non-competitive antagonist	PCP site	No clinical studies due to its significantly increased constitutive neuronal apoptosis in the developing brain [122]	- Despite the neural degeneration found in MK-801 exposure rats, MK-801 is a well-known neuroprotectant in disease models of stroke, traumatic brain injury, and Parkinsonism. - The neuroprotective effects observed in cerebral ischemia or hypoxia are believed to be a result of the inhibition of calcium influx through the blocking of overstimulation of increased glutamate in damaged tissue [123]
	Amantadine	non-competitive antagonist	PCP site	No clinical studies exist currently, and it shows no significant effect in animal SE model [124]	- Amantadine was first approved as an antiviral agent via interfering with viral replication, but was not recommended due to the high resistant rate currently - Amantadine can be used as short-term therapy with levodopa in patients with mild Parkinson’s disease to relieve symptoms - The main advantage is its low side effect profile though livedo reticularis is an uncommon side effect associated with amantadine
	Memantine	non-competitive antagonist	PCP site	No clinical studies now, and it did not prevent the development of SE, while it showed a potential effect of preventing neural death, and reducing cognitive impairment in post-SE condition [114,125]	- Memantine is currently used to slow the neurotoxicity thought to occur in Alzheimer’s disease and other neurodegenerative diseases - Its common side effects of dizziness, headache, and confusion cause discontinuation of therapy
	Dextromethorphan	non-competitive antagonist	PCP site	No clinical studies for SE, while it showed some limited efficacy in refractory seizures as an add-on therapy [116]	- Approved by FDA for cough suppression and pseudobulbar affect - Over the past 2 decades, dextromethorphan has shown evidence of neuroprotective in models of stroke, pain, or traumatic brain injury (TBI) - At high doses, dextromethorphan may precipitate seizures - Potential therapeutic uses in depression, stroke, TBI, seizure, pain, autism
AMPA	Perampanel	non-competitive antagonist	GluA2 subunit	Retrospective clinical studies showed a response rate of around 35% in SE or RSE patients as a combined medication, but further RCT for appropriate timing and dosage is needed	- The most common side effects, including dizziness, and somnolence, which leads to withdrawal from perampanel usage, are dosage dependent - In real-world data in Europe, perampanel as an add-on therapy was continued for at least one year in 48% of patients

## Data Availability

Not applicable.

## References

[1] Eriksson K., Metsäranta P., Huhtala H., Auvinen A., Kuusela A.-L., Koivikko M. (2005). Treatment Delay and the Risk of Prolonged Status Epilepticus. Neurology.

[2] Trinka E., Cock H., Hesdorffer D., Rossetti A.O., Scheffer I.E., Shinnar S., Shorvon S., Lowenstein D.H. (2015). A Definition and Classification of Status Epilepticus—Report of the ILAE Task Force on Classification of Status Epilepticus. Epilepsia.

[3] Nelson S.E., Varelas P.N. (2018). Status Epilepticus, Refractory Status Epilepticus, and Super-Refractory Status Epilepticus. CONTINUUM Lifelong Learn. Neurol..

[4] Wisden W., Seeburg P.H. (1993). Mammalian Ionotropic Glutamate Receptors. Curr. Opin. Neurobiol..

[5] Ahmed H., Haider A., Ametamey S.M. (2020). N-Methyl-D-Aspartate (NMDA) Receptor Modulators: A Patent Review (2015-Present). Expert Opin. Ther. Pat..

[6] Wasterlain C.G., Fujikawa D.G., Penix L., Sankar R. (1993). Pathophysiological Mechanisms of Brain Damage from Status Epilepticus. Epilepsia.

[7] Löscher W. (2007). Mechanisms of Drug Resistance in Status Epilepticus. Epilepsia.

[8] Rajasekaran K., Joshi S., Kozhemyakin M., Todorovic M.S., Kowalski S., Balint C., Kapur J. (2013). Receptor Trafficking Hypothesis Revisited: Plasticity of AMPA Receptors during Established Status Epilepticus. Epilepsia.

[9] Sagratella S. (1995). NMDA Antagonists: Antiepileptic-Neuroprotective Drugs with Diversified Neuropharmacological Profiles. Pharm. Res.

[10] Erdo F., Berzsenyi P., Andrási F. (2005). The AMPA-Antagonist Talampanel Is Neuroprotective in Rodent Models of Focal Cerebral Ischemia. Brain Res. Bull..

[11] Gigler G., Móricz K., Agoston M., Simó A., Albert M., Benedek A., Kapus G., Kertész S., Vegh M., Barkóczy J. (2007). Neuroprotective and Anticonvulsant Effects of EGIS-8332, a Non-Competitive AMPA Receptor Antagonist, in a Range of Animal Models. Br. J. Pharm..

[12] Hendry S.H., Schwark H.D., Jones E.G., Yan J. (1987). Numbers and Proportions of GABA-Immunoreactive Neurons in Different Areas of Monkey Cerebral Cortex. J. Neurosci..

[13] Meldrum B.S. (2000). Glutamate as a Neurotransmitter in the Brain: Review of Physiology and Pathology. J. Nutr..

[14] Dakshinamurti K., Sharma S.K., Sundaram M. (1991). Domoic Acid Induced Seizure Activity in Rats. Neurosci. Lett..

[15] Cendes F., Andermann F., Carpenter S., Zatorre R.J., Cashman N.R. (1995). Temporal Lobe Epilepsy Caused by Domoic Acid Intoxication: Evidence for Glutamate Receptor-Mediated Excitotoxicity in Humans. Ann. Neurol..

[16] Mareš P., Velišek L. (1992). N-Methyl-d-Aspartate (NMDA)-Induced Seizures in Developing Rats. Dev. Brain Res..

[17] Watkins J.C., Olverman H.J. (1987). Agonists and Antagonists for Excitatory Amino Acid Receptors. Trends Neurosci..

[18] Iacobucci G.J., Popescu G.K. (2017). NMDA Receptors: Linking Physiological Output to Biophysical Operation. Nat. Rev. Neurosci..

[19] Mennerick S., Zorumski C.F. (2000). Neural Activity and Survival in the Developing Nervous System. Mol. Neurobiol..

[20] Collingridge G.L., Bliss T.V. (1995). Memories of NMDA Receptors and LTP. Trends Neurosci..

[21] Rison R.A., Stanton P.K. (1995). Long-Term Potentiation and N-Methyl-D-Aspartate Receptors: Foundations of Memory and Neurologic Disease?. Neurosci. Biobehav. Rev..

[22] Ladépêche L., Dupuis J.P., Groc L. (2014). Surface Trafficking of NMDA Receptors: Gathering from a Partner to Another. Semin. Cell Dev. Biol..

[23] Blanke M.L., VanDongen A.M.J., Van Dongen A.M. (2009). Activation Mechanisms of the NMDA Receptor. Biology of the NMDA Receptor.

[24] Kapur J. (2018). Role of NMDA Receptors in the Pathophysiology and Treatment of Status Epilepticus. Epilepsia Open.

[25] Salussolia C.L., Prodromou M.L., Borker P., Wollmuth L.P. (2011). Arrangement of Subunits in Functional NMDA Receptors. J. Neurosci..

[26] Paoletti P. (2011). Molecular Basis of NMDA Receptor Functional Diversity. Eur. J. Neurosci..

[27] Paoletti P., Bellone C., Zhou Q. (2013). NMDA Receptor Subunit Diversity: Impact on Receptor Properties, Synaptic Plasticity and Disease. Nat. Rev. Neurosci..

[28] Hansen K.B., Yi F., Perszyk R.E., Menniti F.S., Traynelis S.F., Burnashev N., Szepetowski P. (2017). NMDA Receptors in the Central Nervous System. NMDA Receptors: Methods and Protocols.

[29] Lipton S.A. (2006). Paradigm Shift in Neuroprotection by NMDA Receptor Blockade: Memantine and Beyond. Nat. Rev. Drug Discov..

[30] Sarlo G.L., Holton K.F. (2021). Brain Concentrations of Glutamate and GABA in Human Epilepsy: A Review. Seizure.

[31] Slais K., Vorisek I., Zoremba N., Homola A., Dmytrenko L., Sykova E. (2008). Brain Metabolism and Diffusion in the Rat Cerebral Cortex during Pilocarpine-Induced Status Epilepticus. Exp. Neurol..

[32] Pollard H., Cantagrel S., Charriaut-Marlangue C., Moreau J., Ben Ari Y. (1994). Apoptosis Associated DNA Fragmentation in Epileptic Brain Damage. Neuroreport.

[33] Leo A., Giovannini G., Russo E., Meletti S. (2018). The Role of AMPA Receptors and Their Antagonists in Status Epilepticus. Epilepsia.

[34] Guo C., Ma Y.-Y. (2021). Calcium Permeable-AMPA Receptors and Excitotoxicity in Neurological Disorders. Front. Neural Circuits.

[35] Man H.-Y. (2011). GluA2-Lacking, Calcium-Permeable AMPA Receptors--Inducers of Plasticity?. Curr. Opin. Neurobiol..

[36] Joshi S., Rajasekaran K., Sun H., Williamson J., Kapur J. (2017). Enhanced AMPA Receptor-Mediated Neurotransmission on CA1 Pyramidal Neurons during Status Epilepticus. Neurobiol. Dis..

[37] Rajasekaran K., Todorovic M., Kapur J. (2012). Calcium-Permeable AMPA Receptors Are Expressed in a Rodent Model of Status Epilepticus. Ann. Neurol..

[38] Fountain N.B. (2000). Status Epilepticus: Risk Factors and Complications. Epilepsia.

[39] Gan J., Qu Y., Li J., Zhao F., Mu D. (2015). An Evaluation of the Links between MicroRNA, Autophagy, and Epilepsy. Rev. Neurosci..

[40] Kapur J., Macdonald R.L. (1997). Rapid Seizure-Induced Reduction of Benzodiazepine and Zn^2+^ Sensitivity of Hippocampal Dentate Granule Cell GABAA Receptors. J. Neurosci..

[41] Mazarati A.M., Baldwin R.A., Sankar R., Wasterlain C.G. (1998). Time-Dependent Decrease in the Effectiveness of Antiepileptic Drugs during the Course of Self-Sustaining Status Epilepticus. Brain Res..

[42] Treiman D.M., Meyers P.D., Walton N.Y., Collins J.F., Colling C., Rowan A.J., Handforth A., Faught E., Calabrese V.P., Uthman B.M. (1998). A Comparison of Four Treatments for Generalized Convulsive Status Epilepticus. Veterans Affairs Status Epilepticus Cooperative Study Group. N. Engl. J. Med..

[43] Lowenstein D.H., Alldredge B.K. (1993). Status Epilepticus at an Urban Public Hospital in the 1980s. Neurology.

[44] Sánchez Fernández I., Goodkin H.P., Scott R.C. (2019). Pathophysiology of Convulsive Status Epilepticus. Seizure.

[45] Kapur J., Coulter D.A. (1995). Experimental Status Epilepticus Alters Gamma-Aminobutyric Acid Type A Receptor Function in CA1 Pyramidal Neurons. Ann Neurol.

[46] Gibbs J.W., Sombati S., DeLorenzo R.J., Coulter D.A. (1997). Physiological and Pharmacological Alterations in Postsynaptic GABA(A) Receptor Function in a Hippocampal Culture Model of Chronic Spontaneous Seizures. J. Neurophysiol..

[47] Goodkin H.P., Yeh J.-L., Kapur J. (2005). Status Epilepticus Increases the Intracellular Accumulation of GABAA Receptors. J. Neurosci..

[48] Naylor D.E., Liu H., Wasterlain C.G. (2005). Trafficking of GABA(A) Receptors, Loss of Inhibition, and a Mechanism for Pharmacoresistance in Status Epilepticus. J. Neurosci..

[49] Mewasingh L.D., Sékhara T., Aeby A., Christiaens F.J.C., Dan B. (2003). Oral Ketamine in Paediatric Non-Convulsive Status Epilepticus. Seizure.

[50] Mazarati A.M., Wasterlain C.G. (1999). N-Methyl-D-Asparate Receptor Antagonists Abolish the Maintenance Phase of Self-Sustaining Status Epilepticus in Rat. Neurosci. Lett..

[51] Yen W., Williamson J., Bertram E.H., Kapur J. (2004). A Comparison of Three NMDA Receptor Antagonists in the Treatment of Prolonged Status Epilepticus. Epilepsy Res..

[52] Van Zundert B., Yoshii A., Constantine-Paton M. (2004). Receptor Compartmentalization and Trafficking at Glutamate Synapses: A Developmental Proposal. Trends Neurosci..

[53] Snyder E.M., Nong Y., Almeida C.G., Paul S., Moran T., Choi E.Y., Nairn A.C., Salter M.W., Lombroso P.J., Gouras G.K. (2005). Regulation of NMDA Receptor Trafficking by Amyloid-Beta. Nat. Neurosci..

[54] Chen N., Luo T., Wellington C., Metzler M., McCutcheon K., Hayden M.R., Raymond L.A. (1999). Subtype-Specific Enhancement of NMDA Receptor Currents by Mutant Huntingtin. J. Neurochem..

[55] Gee C.E., Benquet P., Raineteau O., Rietschin L., Kirbach S.W., Gerber U. (2006). NMDA Receptors and the Differential Ischemic Vulnerability of Hippocampal Neurons. Eur. J. Neurosci..

[56] Naylor D.E., Liu H., Niquet J., Wasterlain C.G. (2013). Rapid Surface Accumulation of NMDA Receptors Increases Glutamatergic Excitation during Status Epilepticus. Neurobiol. Dis..

[57] Lu W., Roche K.W. (2012). Posttranslational Regulation of AMPA Receptor Trafficking and Function. Curr. Opin. Neurobiol..

[58] Lee H.K., Barbarosie M., Kameyama K., Bear M.F., Huganir R.L. (2000). Regulation of Distinct AMPA Receptor Phosphorylation Sites during Bidirectional Synaptic Plasticity. Nature.

[59] Silayeva L., Deeb T.Z., Hines R.M., Kelley M.R., Munoz M.B., Lee H.H.C., Brandon N.J., Dunlop J., Maguire J., Davies P.A. (2015). KCC2 Activity Is Critical in Limiting the Onset and Severity of Status Epilepticus. Proc. Natl. Acad. Sci. USA.

[60] Kelley M.R., Deeb T.Z., Brandon N.J., Dunlop J., Davies P.A., Moss S.J. (2016). Compromising KCC2 Transporter Activity Enhances the Development of Continuous Seizure Activity. Neuropharmacology.

[61] Brophy G.M., Bell R., Claassen J., Alldredge B., Bleck T.P., Glauser T., Laroche S.M., Riviello J.J., Shutter L., Sperling M.R. (2012). Guidelines for the Evaluation and Management of Status Epilepticus. Neurocrit. Care.

[62] Shorvon S., Ferlisi M. (2011). The Treatment of Super-Refractory Status Epilepticus: A Critical Review of Available Therapies and a Clinical Treatment Protocol. Brain.

[63] Strzelczyk A., Ansorge S., Hapfelmeier J., Bonthapally V., Erder M.H., Rosenow F. (2017). Costs, Length of Stay, and Mortality of Super-Refractory Status Epilepticus: A Population-Based Study from Germany. Epilepsia.

[64] Novy J., Logroscino G., Rossetti A.O. (2010). Refractory Status Epilepticus: A Prospective Observational Study. Epilepsia.

[65] Delaj L., Novy J., Ryvlin P., Marchi N.A., Rossetti A.O. (2017). Refractory and Super-Refractory Status Epilepticus in Adults: A 9-Year Cohort Study. Acta Neurol. Scand..

[66] Ferlisi M., Shorvon S. (2012). The Outcome of Therapies in Refractory and Super-Refractory Convulsive Status Epilepticus and Recommendations for Therapy. Brain.

[67] Holtkamp M., Othman J., Buchheim K., Meierkord H. (2005). Predictors and Prognosis of Refractory Status Epilepticus Treated in a Neurological Intensive Care Unit. J. Neurol. Neurosurg. Psychiatry.

[68] Wilder-Smith E.P.V., Lim E.C.H., Teoh H.L., Sharma V.K., Tan J.J.H., Chan B.P.L., Ong B.K.C. (2005). The NORSE (New-Onset Refractory Status Epilepticus) Syndrome: Defining a Disease Entity. Ann. Acad. Med. Singap..

[69] Wickstrom R., Taraschenko O., Dilena R., Payne E.T., Specchio N., Nabbout R., Koh S., Gaspard N., Hirsch L.J. (2022). International NORSE Consensus Group International Consensus Recommendations for Management of New Onset Refractory Status Epilepticus (NORSE) Including Febrile Infection-Related Epilepsy Syndrome (FIRES): Summary and Clinical Tools. Epilepsia.

[70] Devinsky O., Spruill T., Thurman D., Friedman D. (2016). Recognizing and Preventing Epilepsy-Related Mortality: A Call for Action. Neurology.

[71] Massey C.A., Sowers L.P., Dlouhy B.J., Richerson G.B. (2014). Mechanisms of Sudden Unexpected Death in Epilepsy: The Pathway to Prevention. Nat. Rev. Neurol..

[72] Nashef L. (1997). Sudden Unexpected Death in Epilepsy: Terminology and Definitions. Epilepsia.

[73] Devinsky O., Hesdorffer D.C., Thurman D.J., Lhatoo S., Richerson G. (2016). Sudden Unexpected Death in Epilepsy: Epidemiology, Mechanisms, and Prevention. Lancet Neurol..

[74] Buchanan G.F. (2019). Impaired CO2-Induced Arousal in SIDS and SUDEP. Trends Neurosci..

[75] Jansen N.A., Schenke M., Voskuyl R.A., Thijs R.D., van den Maagdenberg A.M.J.M., Tolner E.A. (2019). Apnea Associated with Brainstem Seizures in Cacna1aS218L Mice Is Caused by Medullary Spreading Depolarization. J. Neurosci..

[76] Lertwittayanon W., Devinsky O., Carlen P.L. (2020). Cardiorespiratory Depression from Brainstem Seizure Activity in Freely Moving Rats. Neurobiol. Dis..

[77] Seillier C., Lesept F., Toutirais O., Potzeha F., Blanc M., Vivien D. (2022). Targeting NMDA Receptors at the Neurovascular Unit: Past and Future Treatments for Central Nervous System Diseases. Int. J. Mol. Sci..

[78] Mealing G.A., Lanthorn T.H., Murray C.L., Small D.L., Morley P. (1999). Differences in Degree of Trapping of Low-Affinity Uncompetitive N-Methyl-D-Aspartic Acid Receptor Antagonists with Similar Kinetics of Block. J. Pharmacol. Exp. Ther..

[79] Huettner J.E., Bean B.P. (1988). Block of N-Methyl-D-Aspartate-Activated Current by the Anticonvulsant MK-801: Selective Binding to Open Channels. Proc. Natl. Acad. Sci. USA.

[80] Domino E.F., Chodoff P., Corssen G. (1965). Pharmacologic Effects of CI-581, A New Dissociative Anesthetic, in Man. Clin. Pharm. Ther..

[81] Oranje B., van Berckel B.N., Kemner C., van Ree J.M., Kahn R.S., Verbaten M.N. (2000). The Effects of a Sub-Anaesthetic Dose of Ketamine on Human Selective Attention. Neuropsychopharmacology.

[82] Orser B.A., Pennefather P.S., MacDonald J.F. (1997). Multiple Mechanisms of Ketamine Blockade of N-Methyl-D-Aspartate Receptors. Anesthesiology.

[83] Corriger A., Pickering G. (2019). Ketamine and Depression: A Narrative Review. Drug Des. Dev. Ther..

[84] Liriano F., Hatten C., Schwartz T.L. (2019). Ketamine as Treatment for Post-Traumatic Stress Disorder: A Review. Drugs Context.

[85] Santos E., Olivares-Rivera A., Major S., Sánchez-Porras R., Uhlmann L., Kunzmann K., Zerelles R., Kentar M., Kola V., Aguilera A.H. (2019). Lasting S-Ketamine Block of Spreading Depolarizations in Subarachnoid Hemorrhage: A Retrospective Cohort Study. Crit. Care.

[86] Godoy D.A., Badenes R., Pelosi P., Robba C. (2021). Ketamine in Acute Phase of Severe Traumatic Brain Injury “an Old Drug for New Uses?”. Crit. Care.

[87] Lauritsen C., Mazuera S., Lipton R.B., Ashina S. (2016). Intravenous Ketamine for Subacute Treatment of Refractory Chronic Migraine: A Case Series. J. Headache Pain.

[88] Alkhachroum A., Der-Nigoghossian C.A., Mathews E., Massad N., Letchinger R., Doyle K., Chiu W.-T., Kromm J., Rubinos C., Velazquez A. (2020). Ketamine to Treat Super-Refractory Status Epilepticus. Neurology.

[89] Zeiler F.A., Teitelbaum J., West M., Gillman L.M. (2014). The Ketamine Effect on Intracranial Pressure in Nontraumatic Neurological Illness. J. Crit. Care.

[90] Caranzano L., Novy J., Rossetti A.O. (2022). Ketamine in Adult Super-refractory Status Epilepticus: Efficacy Analysis on a Prospective Registry. Acta Neurol. Scand..

[91] Rosati A., L’Erario M., Ilvento L., Cecchi C., Pisano T., Mirabile L., Guerrini R. (2012). Efficacy and Safety of Ketamine in Refractory Status Epilepticus in Children. Neurology.

[92] Fang Y., Wang X. (2015). Ketamine for the Treatment of Refractory Status Epilepticus. Seizure.

[93] Ilvento L., Rosati A., Marini C., L’Erario M., Mirabile L., Guerrini R. (2015). Ketamine in Refractory Convulsive Status Epilepticus in Children Avoids Endotracheal Intubation. Epilepsy Behav..

[94] Hurth K.P., Jaworski A., Thomas K.B., Kirsch W.B., Rudoni M.A., Wohlfarth K.M. (2020). The Reemergence of Ketamine for Treatment in Critically Ill Adults. Crit. Care Med..

[95] Rosati A., L’Erario M., Bianchi R., Olivotto S., Battaglia D.I., Darra F., Biban P., Biggeri A., Catelan D., Danieli G. (2022). KETASER01 Protocol: What Went Right and What Went Wrong. Epilepsia Open.

[96] Gabbe S.G. (1996). A Preliminary Report on the Intravenous Use of Magnesium Sulphate in Puerperal Eclampsia. 1925. Am. J. Obs. Gynecol..

[97] Hunter L.A., Gibbins K.J. (2011). Magnesium Sulfate: Past, Present, and Future. J. Midwifery Womens Health.

[98] Hallak M. (1998). Effect of Parenteral Magnesium Sulfate Administration on Excitatory Amino Acid Receptors in the Rat Brain. Magnes. Res..

[99] Hallak M., Berman R.F., Irtenkauf S.M., Janusz C.A., Cotton D.B. (1994). Magnesium Sulfate Treatment Decreases N-Methyl-D-Aspartate Receptor Binding in the Rat Brain: An Autoradiographic Study. J. Soc. Gynecol. Investig..

[100] Suter C., Klingman W.O. (1955). Neurologic Manifestations of Magnesium Depletion States. Neurology.

[101] (1995). Which Anticonvulsant for Women with Eclampsia? Evidence from the Collaborative Eclampsia Trial. Lancet.

[102] Zeiler F.A., Matuszczak M., Teitelbaum J., Gillman L.M., Kazina C.J. (2015). Magnesium Sulfate for Non-Eclamptic Status Epilepticus. Seizure.

[103] Tetz L.M., Rezk P.E., Ratcliffe R.H., Gordon R.K., Steele K.E., Nambiar M.P. (2006). Development of a Rat Pilocarpine Model of Seizure/Status Epilepticus That Mimics Chemical Warfare Nerve Agent Exposure. Toxicol. Ind. Health.

[104] Shih T.M. (1990). Anticonvulsant Effects of Diazepam and MK-801 in Soman Poisoning. Epilepsy Res..

[105] Murray T.K., Ridley R.M. (1997). The Effect of Dizocilpine (MK-801) on Conditional Discrimination Learning in the Rat. Behav. Pharm..

[106] Harder J.A., Aboobaker A.A., Hodgetts T.C., Ridley R.M. (1998). Learning Impairments Induced by Glutamate Blockade Using Dizocilpine (MK-801) in Monkeys. Br. J. Pharm..

[107] Kornhuber J., Bormann J., Hübers M., Rusche K., Riederer P. (1991). Effects of the 1-Amino-Adamantanes at the MK-801-Binding Site of the NMDA-Receptor-Gated Ion Channel: A Human Postmortem Brain Study. Eur. J. Pharm..

[108] Wilson R.B., Eliyan Y., Sankar R., Hussain S.A. (2018). Amantadine: A New Treatment for Refractory Electrical Status Epilepticus in Sleep. Epilepsy Behav..

[109] Moryl E., Danysz W., Quack G. (1993). Potential Antidepressive Properties of Amantadine, Memantine and Bifemelane. Pharm. Toxicol..

[110] Xia P., Chen H.V., Zhang D., Lipton S.A. (2010). Memantine Preferentially Blocks Extrasynaptic over Synaptic NMDA Receptor Currents in Hippocampal Autapses. J. Neurosci..

[111] Kabir M.T., Sufian M.A., Uddin M.S., Begum M.M., Akhter S., Islam A., Mathew B., Islam M.S., Amran M.S., Md Ashraf G. (2019). NMDA Receptor Antagonists: Repositioning of Memantine as a Multitargeting Agent for Alzheimer’s Therapy. Curr. Pharm. Des..

[112] Kalemenev S.V., Zubareva O.E., Sizov V.V., Lavrent’eva V.V., Lukomskaya N.Y., Kim K.K., Zaitsev A.V., Magazanik L.G. (2016). Memantine Attenuates Cognitive Impairments after Status Epilepticus Induced in a Lithium-Pilocarpine Model. Dokl. Biol. Sci..

[113] Zaitsev A.V., Kim K.K., Vasilev D.S., Lukomskaya N.Y., Lavrentyeva V.V., Tumanova N.L., Zhuravin I.A., Magazanik L.G. (2015). N-Methyl-D-Aspartate Receptor Channel Blockers Prevent Pentylenetetrazole-Induced Convulsions and Morphological Changes in Rat Brain Neurons. J. Neurosci. Res..

[114] Zenki K.C., Kalinine E., Zimmer E.R., Dos Santos T.G., Mussulini B.H.M., Portela L.V.C., de Oliveira D.L. (2018). Memantine Decreases Neuronal Degeneration in Young Rats Submitted to LiCl-Pilocarpine-Induced Status Epilepticus. Neurotoxicology.

[115] Taylor C.P., Traynelis S.F., Siffert J., Pope L.E., Matsumoto R.R. (2016). Pharmacology of Dextromethorphan: Relevance to Dextromethorphan/Quinidine (Nuedexta^®^) Clinical Use. Pharm. Ther..

[116] Nguyen L., Thomas K.L., Lucke-Wold B.P., Cavendish J.Z., Crowe M.S., Matsumoto R.R. (2016). Dextromethorphan: An Update on Its Utility for Neurological and Neuropsychiatric Disorders. Pharm. Ther..

[117] Kimiskidis V.K., Mirtsou-Fidani V., Papaioannidou P.G., Niopas I., Georgiadis G., Constadinidis T.C., Kazis A.D. (1999). A Phase I Clinical Trial of Dextromethorphan in Intractable Partial Epilepsy. Methods Find. Exp. Clin. Pharm..

[118] Schmitt B., Netzer R., Fanconi S., Baumann P., Boltshauser E. (1994). Drug Refractory Epilepsy in Brain Damage: Effect of Dextromethorphan on EEG in Four Patients. J. Neurol. Neurosurg. Psychiatry.

[119] Zanos P., Moaddel R., Morris P.J., Riggs L.M., Highland J.N., Georgiou P., Pereira E.F.R., Albuquerque E.X., Thomas C.J., Zarate C.A. (2018). Ketamine and Ketamine Metabolite Pharmacology: Insights into Therapeutic Mechanisms. Pharm. Rev..

[120] Euser A.G., Cipolla M.J. (2009). Magnesium Sulfate for the Treatment of Eclampsia: A Brief Review. Stroke.

[121] Lodge D., Mercier M.S. (2015). Ketamine and Phencyclidine: The Good, the Bad and the Unexpected. Br. J. Pharmacol..

[122] Ikonomidou C., Bosch F., Miksa M., Bittigau P., Vöckler J., Dikranian K., Tenkova T.I., Stefovska V., Turski L., Olney J.W. (1999). Blockade of NMDA Receptors and Apoptotic Neurodegeneration in the Developing Brain. Science.

[123] Kovacic P., Somanathan R. (2010). Clinical Physiology and Mechanism of Dizocilpine (MK-801). Oxid. Med. Cell. Longev..

[124] Mohammad H., Taghibiglou C., Moien-Afshari F. (2018). Effect of Perampanel and Amantadine on Rat Model of Pilocarpine-Induced Status Epilepticus: Evidence on Seizure Termination, Behavioral Alterations, Epileptogenesis and Neuronal Damage (P4.268). Neurology.

[125] The NMDA Receptor Channel Blockers Memantine and IEM-1921 Decrease the Duration of Status Epilepticus in Wistar and Krushinskii–Molodkina Rats in a Lithium-Pilocarpine Model|SpringerLink. https://link.springer.com/article/10.1007/s11055-020-00909-y.

[126] Citraro R., Aiello R., Franco V., De Sarro G., Russo E. (2014). Targeting α-Amino-3-Hydroxyl-5-Methyl-4-Isoxazole-Propionate Receptors in Epilepsy. Expert Opin. Ther. Targets.

[127] Goulton C.S., Patten A.R., Kerr J.R., Kerr D.S. (2010). Pharmacological Preconditioning with GYKI 52466: A Prophylactic Approach to Neuroprotection. Front. Neurosci..

[128] Keppel Hesselink J.M. (2017). NS1209/SPD 502, A Novel Selective AMPA Antagonist for Stroke, Neuropathic Pain or Epilepsy? Drug Development Lessons Learned. Drug Dev. Res..

[129] Hanada T., Ido K., Kosasa T. (2014). Effect of Perampanel, a Novel AMPA Antagonist, on Benzodiazepine-resistant Status Epilepticus in a Lithium-pilocarpine Rat Model. Pharmacol. Res. Perspect..

[130] Lim S.-N., Wu T., Tseng W.-E.J., Chiang H.-I., Cheng M.-Y., Lin W.-R., Lin C.-N. (2021). Efficacy and Safety of Perampanel in Refractory and Super-Refractory Status Epilepticus: Cohort Study of 81 Patients and Literature Review. J. Neurol..

[131] Ho C.-J., Lin C.-H., Lu Y.-T., Shih F.-Y., Hsu C.-W., Tsai W.-C., Tsai M.-H. (2019). Perampanel Treatment for Refractory Status Epilepticus in a Neurological Intensive Care Unit. Neurocrit. Care.

[132] Perez D.Q., Espiritu A.I., Jamora R.D.G. (2022). Perampanel in Achieving Status Epilepticus Cessation: A Systematic Review. Epilepsy Behav..

[133] Strzelczyk A., Knake S., Kälviäinen R., Santamarina E., Toledo M., Willig S., Rohracher A., Trinka E., Rosenow F. (2019). Perampanel for Treatment of Status Epilepticus in Austria, Finland, Germany, and Spain. Acta Neurol. Scand..

[134] Glauser T., Shinnar S., Gloss D., Alldredge B., Arya R., Bainbridge J., Bare M., Bleck T., Dodson W.E., Garrity L. (2016). Evidence-Based Guideline: Treatment of Convulsive Status Epilepticus in Children and Adults: Report of the Guideline Committee of the American Epilepsy Society. Epilepsy Curr..

[135] Outin H., Lefort H., Peigne V. (2021). Guidelines, the F.G. for S.E. Guidelines for the Management of Status Epilepticus. Eur. J. Emerg. Med..

[136] Lumley L.A., Marrero-Rosado B., Rossetti F., Schultz C.R., Stone M.F., Niquet J., Wasterlain C.G. (2021). Combination of Antiseizure Medications Phenobarbital, Ketamine, and Midazolam Reduces Soman-induced Epileptogenesis and Brain Pathology in Rats. Epilepsia Open.

[137] Niquet J., Lumley L., Baldwin R., Rossetti F., Schultz M., de Araujo Furtado M., Suchomelova L., Naylor D., Franco-Estrada I., Wasterlain C.G. (2019). Early Polytherapy for Benzodiazepine-Refractory Status Epilepticus. Epilepsy Behav..

